# A Critical Review of ChatGPT as a Potential Substitute for Diabetes Educators

**DOI:** 10.7759/cureus.38380

**Published:** 2023-05-01

**Authors:** Samriddhi Sharma, Sandhya Pajai, Roshan Prasad, Mayur B Wanjari, Pratiksha K Munjewar, Ranjana Sharma, Aniket Pathade

**Affiliations:** 1 Medicine and Surgery, Jawaharlal Nehru Medical College, Datta Meghe Institute of Higher Education and Research, Wardha, IND; 2 Obstetrics and Gynaecology, Jawaharlal Nehru Medical College, Datta Meghe Institute of Higher Education and Research, Wardha, IND; 3 Research and Development, Jawaharlal Nehru Medical College, Datta Meghe Institute of Higher Education and Research, Wardha, IND; 4 Medical Surgical Nursing, Srimati Radhikabai Meghe Memorial College of Nursing, Datta Meghe Institute of Higher Education and Research, Wardha, IND

**Keywords:** ethical considerations, emotional support, medical advice, patient engagement, healthcare communication, health technology, patient education, artificial intelligence, diabetes education, chatgpt

## Abstract

This review article explores the potential of ChatGPT as a substitute for diabetes educators. Diabetes is a prevalent chronic disease that requires ongoing education and support for patients to effectively manage their condition. However, there is a shortage of diabetes educators, and traditional education methods have limitations in addressing patients' individual needs. ChatGPT is an artificial intelligence technology that offers a personalized and interactive approach to education and support. In this review, we provide an overview of ChatGPT technology, discuss the challenges facing diabetes educators, review evidence supporting the use of ChatGPT in diabetes education, and examine ethical considerations related to its use. We also provide recommendations for further research and development of ChatGPT in diabetes education and integration into clinical practice. ChatGPT has the potential to improve access to education and support for patients with diabetes, but further research is needed to better understand its effectiveness and limitations. It is important to ensure that ChatGPT is developed and integrated in an ethical and equitable manner to maximize its potential benefits and minimize potential risks.

## Introduction and background

Diabetes is a prevalent chronic disease affecting millions worldwide [1]. Effective management of diabetes necessitates a combination of lifestyle modifications, medication, and regular monitoring of blood glucose levels [2]. Diabetes education is critical in assisting patients in comprehending their condition, managing their symptoms, and improving their quality of life. Diabetes educators are healthcare professionals who provide patients with the necessary information, skills, and support to self-manage their diabetes, including medication management, blood glucose monitoring, and lifestyle changes [1,2].

Artificial intelligence (AI) in healthcare is rapidly increasing, potentially revolutionizing the field [3]. AI-powered tools can help healthcare professionals improve patient care, diagnosis, and treatment outcomes. One area where AI is being increasingly utilized is patient education and support. Chatbots and other AI-powered tools can provide patients with real-time, personalized, and interactive education and support, improving patient engagement and outcomes [4,5].

This review article explores the potential of ChatGPT, a state-of-the-art language model developed by OpenAI, as a substitute for diabetes educators. We will examine the advantages and limitations of using ChatGPT in diabetes education and evaluate its effectiveness compared to traditional diabetes education methods. Additionally, we will discuss ethical considerations related to using ChatGPT in healthcare and identify areas for further research and development. Furthermore, we discuss the limitations and challenges of using ChatGPT as a substitute for diabetes educators.

These limitations include the lack of human interaction and potential biases and inaccuracies in the information provided. We also emphasize the ethical considerations of ChatGPT use, including the importance of patient privacy and confidentiality. Overall, the use of ChatGPT in diabetes education has the potential to improve access to education and support for patients with diabetes. However, further research is needed to understand its effectiveness and limitations better. Ensuring that ChatGPT is developed and integrated ethically and equitably is crucial to maximize its benefits and minimize potential risks.

## Review

What is ChatGPT?

ChatGPT is a cutting-edge language model created by OpenAI, which leverages advanced machine learning algorithms to produce responses to text-based inquiries that mimic human-like conversation [6]. This state-of-the-art natural language processing tool represents one of the most advanced language models currently available. Its exceptional ability to comprehend and answer intricate queries generates coherent and contextually appropriate responses [7]. ChatGPT's ability to produce natural language is accomplished through its extensive training on large-scale text datasets, allowing it to capture and replicate the intricacies and nuances of human language. As a result, ChatGPT can simulate an authentic conversation with remarkable accuracy, leading to its emergence as a promising technology in healthcare, including its application in diabetes education [6,8].

How does ChatGPT work?

ChatGPT operates on a neural network architecture that employs unsupervised learning to analyze copious amounts of text data, enabling it to recognize patterns and relationships between words and phrases [9]. By utilizing this knowledge, ChatGPT can generate human-like responses to text-based queries. The technology is trained on a diverse range of text sources such as books, articles, and online forums, which provides it with a comprehensive understanding of various perspectives and experiences. As a result, ChatGPT can offer responses informed by many sources, enhancing its ability to provide personalized and accurate information [10,11].

Advantages of using ChatGPT in diabetes education

ChatGPT offers several advantages that make it a promising tool for diabetes education. One of its key advantages is providing patients with real-time, personalized education and support. This personalized approach can improve patient engagement and adherence to treatment plans by tailoring the educational content and support to each patient's specific needs and preferences. ChatGPT can also help patients understand complex medical information by conversationally providing clear and concise explanations [12].

Another advantage of ChatGPT is its accessibility. Patients can access it anytime and anywhere, making it a convenient and flexible option. This accessibility can help patients stay engaged in their diabetes management and reduce the likelihood of missed appointments or important information [13].

Furthermore, ChatGPT can be trained on a wide range of text sources, including medical literature and patient forums, which allows it to provide patients with a broad range of information and perspectives. This can help patients make informed decisions about their treatment plans and better understand their condition [13].

In addition, ChatGPT can potentially address the shortage of diabetes educators. With the increasing prevalence of diabetes, there is a growing need for diabetes education and support. However, there is a shortage of diabetes educators, making it challenging for patients to access the necessary education and support. ChatGPT can potentially fill this gap by providing personalized education and support to patients at scale.

The current state of diabetes education

Challenges Facing Diabetes Educators

Diabetes educators encounter numerous challenges in their practice. Among these, a primary obstacle is the limited availability of time and resources to provide customized education and support to each patient. Moreover, many patients, particularly those residing in rural or underserved areas, may have limited access to diabetes education programs, exacerbating this challenge. Additionally, patients may face difficulties adhering to their treatment plans due to a lack of motivation or a poor understanding of their condition. These challenges underscore the critical need for innovative approaches to diabetes education and support, particularly those that can provide personalized and interactive assistance to patients in overcoming these obstacles [14-16].

Limitations of Traditional Diabetes Education Methods

Traditional methods of diabetes education, such as group classes or one-on-one counseling sessions, possess several limitations. These approaches are often time-consuming, making them impractical for patients with busy schedules. Additionally, they may not be accessible to all patients, particularly those who live in remote or rural areas. Moreover, these methods may not provide patients with personalized education and support tailored to their unique needs and preferences. Consequently, patients may not receive the most effective education and support to help them manage their diabetes. Finally, traditional methods may not keep up with the latest research and developments in diabetes management, potentially providing patients with outdated or ineffective education and support [17-20].

Potential Role of ChatGPT in Addressing These Challenges

The potential role of ChatGPT in addressing the challenges faced by diabetes educators and patients is significant. ChatGPT can provide patients with real-time, personalized education and support, which can improve patient engagement and adherence to treatment plans. This personalized approach is particularly relevant to managing diabetes, as patients often require tailored education and support to manage their unique symptoms and comorbidities [21-23].

Furthermore, ChatGPT's accessibility and convenience make it an attractive option for patients facing barriers to accessing traditional diabetes education methods. Patients can access ChatGPT anytime and anywhere, eliminating the need for in-person appointments and travel. This can be particularly beneficial for individuals living in remote areas or with limited mobility [21-23].

Another advantage of ChatGPT is its ability to be trained on various text sources, including medical literature and patient forums. This enables ChatGPT to provide patients with up-to-date and relevant information on diabetes management and treatment options. By staying current with the latest research and best practices, ChatGPT can provide patients with accurate and effective education and support [3,11,14,19,22].

Effectiveness of ChatGPT as a substitute for diabetes educators

Evidence Supporting the Use of ChatGPT in Diabetes Education

Several studies have shown promising results regarding the effectiveness of ChatGPT in diabetes education. For example, a study published in the Journal of Medical Internet Research found that patients who received diabetes education from a ChatGPT system showed significantly improved diabetes self-management behaviors compared to a control group [24].

Comparison of Outcomes Between Traditional Diabetes Education and ChatGPT-Based Education

While studies have shown promising results for ChatGPT-based diabetes education, it is important to compare these outcomes to traditional diabetes education methods. Some studies have found that traditional diabetes education methods, such as group classes and one-on-one counseling, can improve patient outcomes. However, traditional methods may not be as accessible or convenient as ChatGPT-based education and may not provide patients with personalized education and support. Additionally, traditional methods may be unable to keep up with the latest research and developments in diabetes management, which can lead to outdated or ineffective education and support. Overall, while both traditional and ChatGPT-based diabetes education methods can be effective, ChatGPT may offer several advantages regarding accessibility, convenience, and personalization [8,12-14].

Limitations and Challenges to Using ChatGPT in Diabetes Education

Although ChatGPT exhibits potential as a substitute for diabetes educators, several limitations and challenges must be addressed before widespread adoption. One of the primary challenges is ensuring the ChatGPT system's accuracy and reliability, especially when providing medical advice and information. The system's accuracy is paramount to ensure patient safety, and the technology must be continuously updated and validated to maintain its precision. ChatGPT may not provide the same emotional support and empathy as a human diabetes educator, which can be vital for some patients' well-being. Thus, ChatGPT could be a useful supplement to traditional diabetes education methods but may not replace human interaction entirely.

Furthermore, the effectiveness of ChatGPT-based education may be limited by patient comfort and familiarity with technology. While younger generations may be comfortable using technology for diabetes education and support, older patients may be less inclined to use it. Additionally, the ChatGPT system's effectiveness may depend on the patient's digital literacy and their ability to understand and navigate the system. Therefore, it is important to ensure that ChatGPT-based education is offered alongside traditional methods, allowing patients to choose the mode of education that suits them best [21-23].

Finally, there are ethical considerations related to using ChatGPT for diabetes education. Privacy and confidentiality of patient data must be strictly enforced to prevent any unauthorized disclosure of sensitive health information. Additionally, bias and discrimination must be avoided by ensuring that the ChatGPT system is developed using diverse patient data sets and trained with unbiased algorithms. Ensuring ethical considerations are addressed is paramount to ensure the ChatGPT system's safety and efficacy [25-27].

Ethical considerations

Importance of Ethical Considerations in the Use of ChatGPT for Diabetes Education

As with any new technology in healthcare, it is important to consider the ethical implications of using ChatGPT for diabetes education. Patients rely on accurate and trustworthy information and advice when managing their health. Using ChatGPT in this context raises important ethical considerations, such as patient safety, privacy, and autonomy. It is important to ensure that ChatGPT systems are designed and implemented to prioritize patients' best interests and uphold ethical principles [27].

Potential Risks and Benefits for Patients

The use of ChatGPT in diabetes education has potential benefits for patients, such as increased accessibility to personalized education and support and improved outcomes. However, it is also important to consider the potential risks associated with using ChatGPT. For example, inaccurate or inappropriate medical advice provided by the ChatGPT system could lead to negative health outcomes for patients. Moreover, patients may not feel comfortable discussing sensitive or personal health information with a machine, which could lead to reduced trust and engagement with the ChatGPT system. It is important to weigh the potential risks and benefits of ChatGPT-based diabetes education and take steps to mitigate any potential harm [25,26].

Ensuring Patient Privacy and Confidentiality

Another important ethical consideration in using ChatGPT for diabetes education is patient privacy and confidentiality. Patients may share sensitive health information with the ChatGPT system, and it is important to ensure that this information is kept confidential and secure. It is important to have appropriate measures in place to protect patient data, such as data encryption, secure storage, and appropriate access controls. Additionally, patients should be fully informed about how their data will be used and who will have access to it. They should have the opportunity to provide informed consent for using their data. Ensuring patient privacy and confidentiality is essential for building trust and maintaining ethical standards in using ChatGPT for diabetes education [28-30].

Future directions and recommendations

Areas for Further Research and Development of ChatGPT in Diabetes Education

While there is promising evidence for using ChatGPT in diabetes education, there is still much to learn about how this technology can best support patients. Further research is needed to explore the potential benefits and limitations of ChatGPT in different populations, such as those with low health literacy, non-English speakers, and comorbidities. In addition, more studies are needed to evaluate the effectiveness of ChatGPT-based diabetes education in real-world settings and to compare its effectiveness to traditional methods. Further development of ChatGPT systems is also needed to enhance the accuracy and reliability of these systems.

Considerations for Integrating ChatGPT into Clinical Practice

As ChatGPT technology continues to develop and improve, it is important to consider how it can be integrated into clinical practice in an effective and ethical way. Healthcare providers should be trained in how to use ChatGPT technology and how to communicate with patients who are using these systems effectively. It is also important to consider how ChatGPT can be integrated into existing healthcare systems, such as electronic health records, to ensure that patient data is managed appropriately and that healthcare providers have access to relevant information.

Potential Role of ChatGPT in Improving Access to Diabetes Education for Underserved Populations

One potential benefit of ChatGPT in diabetes education is its potential to improve access to education for underserved populations. For example, patients who live in rural or remote areas may have limited access to diabetes educators or may face barriers to attending in-person education sessions. ChatGPT-based education can be accessed from anywhere with an internet connection, potentially increasing access to education and support for these populations. ChatGPT-based education may be particularly helpful for those with lower health literacy, who may struggle to understand complex medical information presented in traditional education settings. However, it is important to ensure that the use of ChatGPT does not exacerbate existing health disparities and that the technology is developed and implemented in a way that is equitable and accessible to all.

## Conclusions

The potential of ChatGPT to support diabetes education has significant implications for the future of diabetes care. With the increasing prevalence of diabetes and the shortage of diabetes educators, ChatGPT technology could provide a much-needed solution to improve access to education and support for patients. By addressing the limitations of traditional education methods and offering a personalized, interactive approach, ChatGPT can potentially improve patient outcomes and satisfaction with diabetes education. While ChatGPT technology shows promise in supporting diabetes education, further research is needed to understand its effectiveness and limitations better. Healthcare providers and policymakers must ensure that the technology is developed and integrated ethically and equitably and that patient privacy and confidentiality are maintained. To fully realize the potential of ChatGPT in diabetes education, it is important to continue to explore and develop this technology while also ensuring that patients receive the support and education, they need to manage their diabetes effectively.
